# Long-Lasting Examinations of Surface and Structural Properties of Medical Polypropylene Modified with Silver Nanoparticles

**DOI:** 10.3390/polym11122018

**Published:** 2019-12-05

**Authors:** Magdalena Ziąbka, Michał Dziadek

**Affiliations:** 1 Department of Ceramics and Refractories, Faculty of Materials Science and Ceramics, AGH University of Science and Technology, 30-059 Krakow, Poland; 2 Department of Glass Technology and Amorphous Coatings, Faculty of Materials Science and Ceramics, AGH University of Science and Technology, 30-059 Krakow, Poland; dziadek@agh.edu.pl; 3 Faculty of Chemistry, Jagiellonian University, 30-387 Krakow, Poland

**Keywords:** polypropylene, silver nanoparticles, composites, surface and structural properties

## Abstract

Composite materials based on polypropylene modified with silver nanoparticles (PP/AgNPs) were manufactured using injection molding and extrusion. Two different matrices were used to prepare the samples consisting of 0.5 and 1.0 wt. % of silver nanoparticles, respectively. The aim of this study was to assess whether silver nanoparticles (AgNPs) could influence the stability of a polymer matrix during the 24-month period of the in vitro testing. The results indicated that composites with silver nanoparticles displayed the significantly higher Young modulus and tensile strength after the first and second year of investigation. Moreover, the incorporation of nanoparticles into the matrix slightly increased the roughness and contact angle values and the parameters remained stable after the in vitro incubation. The two-year immersion of materials in the deionized water proved that the microstructure of composites did not change. The DSC analysis revealed that the material incubation resulted in a slight reduction in the melting temperature and degree of crystallinity of PP. The addition of nanoparticles to polymer matrices led to the increase in content of β crystals in the crystalline phase of PP, which was revealed in the long-term in vitro tests. The XRD measurement also showed the heightened surface crystallinity. The conducted studies have proved that all composites are stable over a period of 24 months. Such behavior suggests that the tested materials can be used as biomaterials.

## 1. Introduction

Polypropylene (PP) is a thermoplastic polymer which is commonly used in plastics industry. It is endowed with the remarkable chemical resistance, efficient mechanical properties, high heat distortion temperature, excellent rigidity, electrical insulation, significant resistance to folding, and ease of molding [1]. Thanks to the low costs of production, PP is often used in many areas of industry, among others in: automotive industry, domestic consumption as packaging films, as industrial products or paints, storage systems, toys, sports equipment, paintbrushes and garden furniture [2,3]. The disadvantages of polypropylene include poor toughness and mold shrinkage [4]. Polypropylene has a crystalline structure with a high level of stiffness and a high melting point, when compared to other commercial thermoplastics. PP is also often chosen as a matrix for composite materials with various modifiers and reinforcements, such as: glass and carbon fibres, carbon nanotube, mica, talk calcium carbonate, wollastonite and silica. Additives are applied to change or improve physicochemical properties, to increase tensile or flexural strength and the Young modulus, to creep resistance or deflection temperature [5]. Some of this fillers are often used to reduce final material costs as well. Polypropylene is popular in a medical area as it offers the hot filling capability and provides a good contact clarity [6]. Medical applications of this polymer include syringes, vials, containers for pills and capsules, jars or bottles for syrups [7]. As a constituent of composite materials, polypropylene modified with nanometals is used to obtain removable prostheses, surgical masks, diapers, filters, burn and wound dressings, and hygienic bands [8,9]. All of these medical devices mostly belong to the first or second class of risk according to the Food and Drug Administration regulations [10]. Polymer implants modified with silver nanoparticles and intended for a long-term contact with the human body belong to the third risk class. The long-term implantation of a medical device (a prosthesis) may lead to the chemical or biological degradation of the material, the loss of mechanical properties, and the matrix degradation, resulting in the loss of the material inertia. Polypropylene is currently the most common material used in the stress urinary incontinence treatment, in breast implants and mesh implants for pelvic floor disorder (PFD) [11]. Clavé et al. [12] showed that synthetic implants used in a vaginal approach for pelvic floor reinforcement degraded more when an acute infection or chronic inflammation took place. One of possible explanations why the polymer structure degrades might be the diffusion of organic molecules (e.g., fatty acids or cholesterol) into the polymer. The second reason is the formation of free radicals that appear during infection and inflammation. When radical oxidation occurs in the absence of oxygen, the radicals may promote cross-linking, which alters the physical and mechanical properties of the polymer. Pikaart et al. also indicated that polypropylene mesh degradation required removing the sling portion that eroded into the bladder [13]. Whereas, Lee et al. convinced that trans-vaginal repair of an anterior vaginal wall prolapse with the monofilament polypropylene mesh is an effective and safe procedure [14]. Achtariet al. [15] compared the erosion rates of two prostheses: a polypropylene nonabsorbable, monofilament, macroporous mesh and a composite polypropylene/polyglactin 910, mono/multifilament, absorbable/nonabsorbable mesh. They found no statistically significant differences between those two prostheses during the first six post-operative months. However, it was revealed that the mesh erosion was also dependent on the surgeon’s experience and the patient age. On the contrary, Prudente et al. [16] demonstrated that the implantation of monofilament and macroporous polypropylene in the subcutaneous of rats resulted in the increased inflammatory activity and higher TNF production in the early post implantation phase. After 30 days, PP had the similar cytokines immune reactivity, vessel density and extracellular matrix organization values. Recently, PP composites filled with Ag or Cu nanoparticles have been studied due to their antibacterial properties [17,18]. Using the spinning process, Yeo and Jeong [19] have prepared sheath—core type nanocomposite fibers using PP chips and PP master-batches with different concentrations of silver nanoparticles—and proved that fibers with silver in the sheath part showed excellent antibacterial effects even in the low silver content. Moreover, they showed that the silver content in nanocomposites decreased crystallinity with no impact on the fusion temperature. Oliani et al. [20] obtained PP/AgNPs nanocomposites by the extrusion process and evaluated some physicochemical and biological properties. They proved that introduction of AgNPs into PP led to a significant decrease of its tensile strength at the nanoparticles content of 1%. In addition, composites enriched with nanosilver showed biocide activity to Gram-positive and Gram-negative bacteria without cytotoxic effect on mouse cells. Jokar et al. [21] also proved antimicrobial efficiency of silver LDPE nanocomposite. However, they pointed out that a high level of silver nanoparticles may lead to weakening of mechanical properties. Furthermore, they paid attention to the thermal stability of the active component and the chemical compatibility of polymer matrix and antimicrobials in order to evenly distribute antimicrobial substance. In spite of all the a fore mentioned data, little information is still available in the literature describing the structure and mechanical properties of the PP composites filled with Ag nanoparticles in a long period observation, as silver nanoparticles may also accelerate the degradation process.

The above literature data indicate the need of conducting research on the behavior of polypropylene matrix for the longest possible period. Therefore, the aim of this article is to determine whether the polypropylene modified with silver nanoparticles may maintain the physicochemical properties without signs of degradation during the long-lasting performance. To study the stability, we characterized two types of polymer matrices modified with different silver contents after the 12 and 24 months of incubation engaging the DSC, XRD, and SEM examinations. We used a tensile tester to investigate the mechanical properties of the composite materials and evaluated the surface properties.

## 2. Materials and Methods

### 2.1. Material Manufacturing

**Figure 1:**
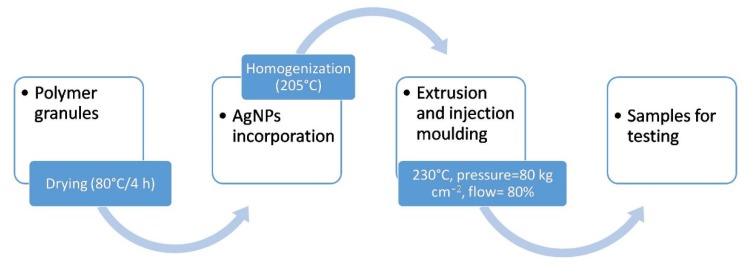
Scheme of samples fabrication.

Two commercially available polymers PP polypropylene- EltexMED 100 (INEOS Olefins and Polymers Europe, Koln, Germany), marked as MG03 (*MFR* = 3 g 10 min^−1^, *E* = 1450 MPa) and MG12 (*MFR* = 12 g 10 min^−1^, *E* = 1400 MPa) and composites modified with 0.5 and 1.0 wt. % nanosilver (NanoAmor, Katy, TX, USA) with a purity of 99.9%, 80 nm particle size and density of 10.49 g cm^−3^ were formed as discs of 10 mm in diameter and paddles. All the materials were fabricated via extrusion and injection molding (Figure 1). The procedure of obtaining specimens consisted of a few steps. First, the granulates were prepared and dried in the laboratory dryer at 80 °C for 4 h. Then, the silver nanoparticles were incorporated and homogenized with polymer granules in the plasticizing chamber using 0.8 m length screw (the homogenization temperature 205 °C). Subsequently, the material was injected into the steel molding form, cooled and extracted. The processing parameters were selected and adapted to the manufacturing process according to characteristic data sheet of each polymer (injection temperature in three zones was 230 °C, injection pressure—80 kg cm^−2^, flow—80%).

### 2.2. Material Evaluation

The obtained materials were incubated in deionized water at the temperature of 37 ± 1 °C for 12 and 24 months. The sample weight to incubation medium ratio equaled 1g:10 mL in accordance with the ISO 10993-13:2010 standard: Identification and quantification of degradation products from polymeric medical devices [22]. All tests were conducted before and after the incubation.

All tests carried out in this work have been selected to enable the assessment of structural and surface properties of the investigated materials. The authors focused primarily on the evaluation of the mechanical properties of polymers and composites measured during a 24-month incubation in water and structural studies. The selection of individual methods, such as XRD, DSC and SEM, confirmed the stable behavior of materials over time, which is extremely important from the point of view of biological stability in medical applications. In addition, studies were carried out to assess surface parameters, such as, wettability and roughness, which are relevant to the interaction of materials in contact with living organisms.

#### 2.2.1. Scanning Electron Microscopy

Detailed microstructure examinations of the materials were carried out using the Nova NanoSEM 200 scanning electron microscope (FEI, Eindhoven, The Netherlands) equipped with a Genesis XM X-ray microanalysis system (EDAX, Tilburg, The Netherlands) featuring the EDAX Sapphire Si(Li) EDX detector. The SEM evaluation was performed in high vacuum conditions, with the back scatter electron detector (BSE) of the accelerated voltage of 10–18 kV. The samples were coated with a carbon layer.

#### 2.2.2. Roughness

The arithmetical mean roughness (Ra) of the MG03, MG12 and their composites with nanosilver was measured using a contact profilometer HOMMEL-ETAMIC T1000 wave (Jenoptik AG, Jena, Germany). The arithmetical mean roughness values were determined as an average of 10 measurements and were expressed as the mean ± standard deviation (SD).

The laser confocal microscope Lext 4000 (Olympus, Tokyo, Japan) with the magnification 50× was used to visualize the surface topography of the MG03, MG12 and their composites with nanosilver. The scanned area was 2.56 mm × 2.56 mm.

#### 2.2.3. Surface Wettability

The static water contact angle was measured by the sessile drop method with an automatic drop shape analysis system DSA 10 Mk2 (Kruss GmbH, Hamburg, Germany). Ultra high quality-water droplets of 0.25 μL were applied on each pure and dry sample. The measurements were performed at a constant temperature in stable humidity conditions. The apparent contact angle was calculated as an average of 10 measurements and expressed as a mean ± standard deviation (SD).

#### 2.2.4. Tensile Test

The tensile strength (*σ*_M_) and Young modulus (*E*_t_) were evaluated using a universal testing machine Inspect Table Blue 5 kN with 5 kN load cell (Hegewald&Peschke, Nossen, Germany). The pre-load force was 1 N, the test speed was 50 mm min^−1^. The investigations were carried out according to the EN ISO 527-1 standards [23]. The mechanical parameters were calculated by averaging 10 measurements and were expressed as a mean ± standard deviation (SD).

#### 2.2.5. Differential Scanning Calorimetry

For the DSC measurements the Netzsch STA 449F3 Jupiter (Netzsch-Gerätebau GmbH, Selb, Germany) was employed. The samples of ca. 4 mg weight were placed in sealed aluminum pans. The heating rate of 10 °C·min^−1^ was applied. Argon was used as an inert gas with the flow rate of 40 mL·min^−1^. Prior to its use, the calorimeter was calibrated with a sapphire standard and an empty aluminum pan was used as a reference. The melting temperature (*T*_m_) was determined at the maximum of the melting endotherm during a single heating run. The degree of crystallinity (*χ*_c_) of PP was estimated using the enthalpy of melting change according to the Equation:

(1) $$\chi_{c}=\frac{\Delta H_{m}}{\left(1-x\right)\Delta H^{{}^{\circ}}{}_{m}}$$

where Δ*H*_m_ and Δ*H*°_m_ are the enthalpy of melting of the sample and the enthalpy of melting of fully crystalline PP (207 J g^−1^), respectively, and *x* is the weight fraction of the AgNPs.

#### 2.2.6. X-ray Diffraction Measurement

The phase analysis of the materials was examined with the XRD, using the X-ray diffractometer (XRD, PhilipsX’Pert Pro, PANanalytical, Almelo, The Netherlands) operating at 50 kV and 30 mA with a CuKa radiation source, in the 2*θ* 10–50° range. Having separated X-ray diffraction lines, the content of the β form (parameter *k*_β_) was calculated using the well-known Turner-Jones Equation [24]:

(2) $$k\beta =\frac{I\beta 1}{I\beta 1+I\alpha 1+I\alpha 2+I\alpha 3}\times 100\%$$

where: *I*α1, *I*α2, *I*α3—the intensities of three typical peaks of *α* form assigned to the (110), (040) and (130) planes of the α cell, respectively, *I*β1—the intensity of the strongest diffraction peak of the form signed to the (300) plane at *d* = 5.495 Å.

## 3. Results

**Figure 2:**
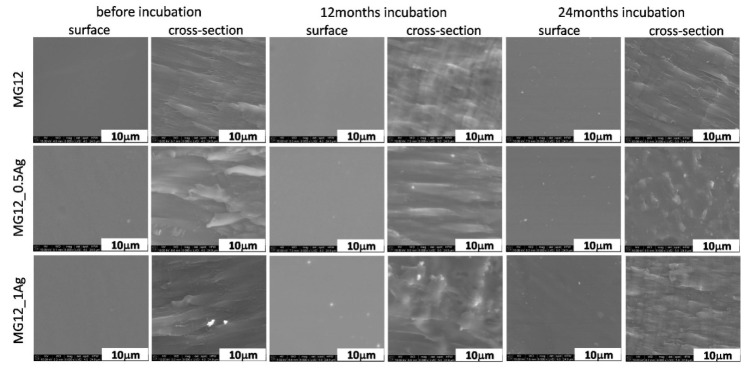
SEM images of surface and cross-section of pure polymer (MG12) and polymer containing 0.5 and 1.0 wt. % of silver nanoparticles (AgNPs) before and after 12 and 24 months of incubation.

**Figure 3:**
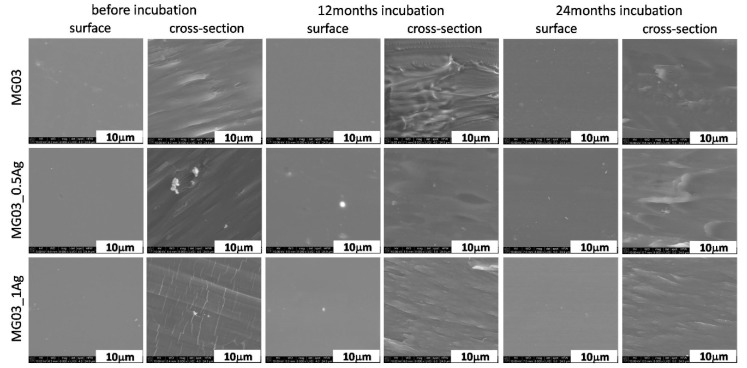
SEM images of surface and cross-section of pure polymer (MG03) and polymer containing 0.5 and 1.0 wt. % of silver nanoparticles (AgNPs) before and after 12 and 24 months of incubation.

The polymer surfaces were homogeneous and smooth, which was proven via the microscopic tests performed on the MG03 (Figure 2) and MG12 (Figure 3) polymers. The surface studies in the aquatic environment after 12 and 24 months of incubation showed no significant changes. This characteristic behavior was observed for the two polymer matrices. The analysis of polymer cross-sections also confirmed the lack of changes in the microstructure of these materials. No cracks or fissures appeared that could indicate the degradation of polymer matrices. Regarding the composites modified with silver nanoparticles, a high degree of homogeneity of silver nanoparticles was observed for both 0.5 and 1 wt. % AgNPs content. However, during the cross-sections observations there were areas where silver nanoparticle agglomerates appeared and such agglomerates were more common in composites containing 1 wt. % AgNPs. During the surface observations after 12 and 24 months of incubation, silver nanoparticles located near the surface were noted, which correlated with the results of XRD tests and the content of the crystalline phase β (Figure 8 and Table 1). When observing the cross-sections after 12 and 24 months incubation, silver nanoparticle agglomerates were also noted, yet the composites microstructure did not change.

**Figure 4:**
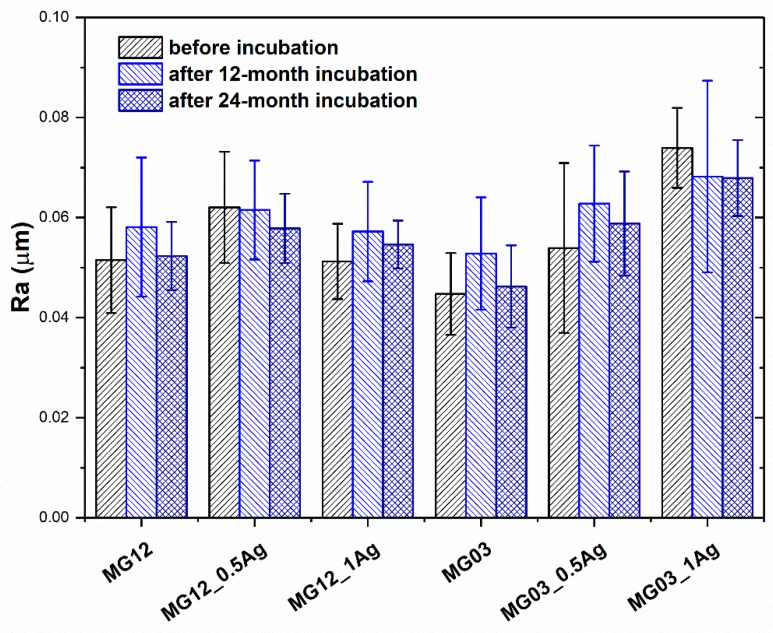
The arithmetical mean roughness (Ra) of pure polymers and polymers containing 0.5 wt. % and 1.0 wt. % of silver nanoparticles (AgNPs).

The introduction of silver nanoparticles into the MG12 polypropylene matrix in the amount of either 0.5 or 1.0 wt. % did not change the roughness parameter (Figure 4). In the case of the MG03 matrix, the 1.0 wt. % concentration of nanoparticles resulted in a significant (40%) increase in roughness. In the case of the output samples, the Ra parameter value was 0.045 μm, while the 1 wt. % content increased the Ra value to 0.074 μm. Furthermore, the 3D visualization via laser confocal microscopy showed uniform and homogeneous surfaces of all the tested materials (Figure S1, from the supplementary material). For all the samples, no change in roughness was noted during the incubation. The absence of changes in the samples’ surface topography after a 24-month incubation in deionized water was also confirmed by laser confocal microscopy. The in vitro tests carried out in distilled water after 12 and 24 months confirmed the constant Ra parameter in comparison to the initial samples. This behavior of materials proved the stability of both polymer matrices over time.

**Figure 5:**
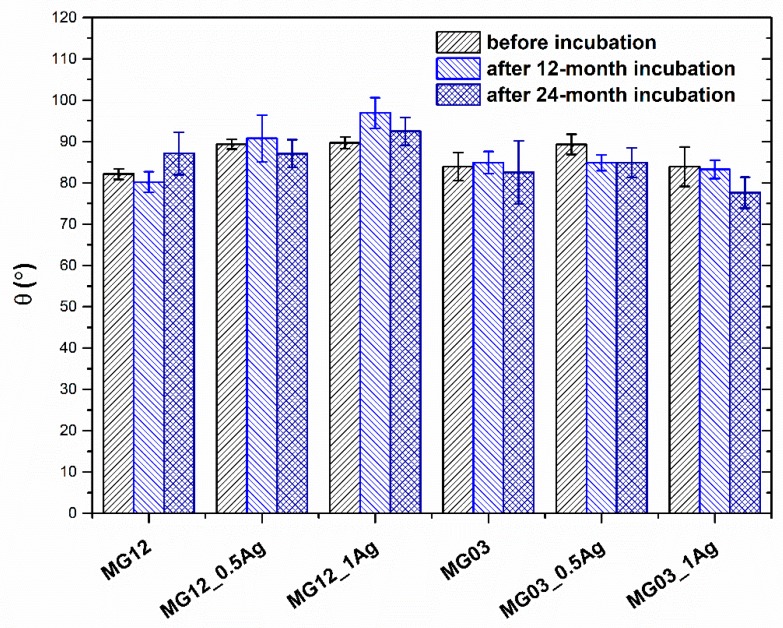
Static water contact angle of pure polymers and polymers containing 0.5 and 1.0 wt. % of silver nanoparticles AgNPs.

The wettability tests conducted on both types of polymers and their composites (Figure 5) showed an increase in the contact angle with an increasing proportion of the modifying phase. The average contact angle for the MG12 polymer matrix equaled about 82° and for the MG03 matrix—about 84°. The introduction of 0.5 and 1.0 wt. % silver nanoparticles caused an increase in the angle to 89° and 90°, and no change for the composites based on MG12 and MG03 matrix, respectively. However, the contact angle tests after 12 and 24 months incubation were similar with no increase in this parameter noted.

**Figure 6:**
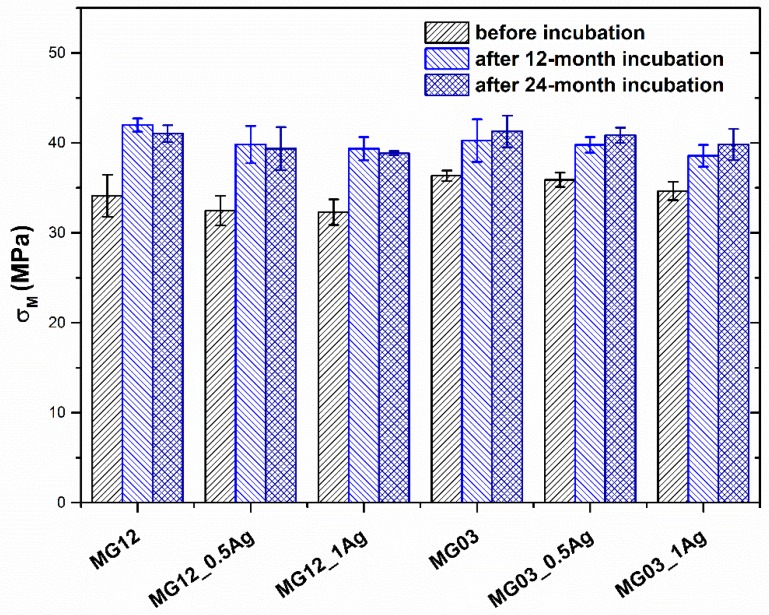
Strength (*σ*_M_) of pure polymers and polymers containing 0.5 and 1.0 wt. % of silver nanoparticles (AgNPs).

**Figure 7:**
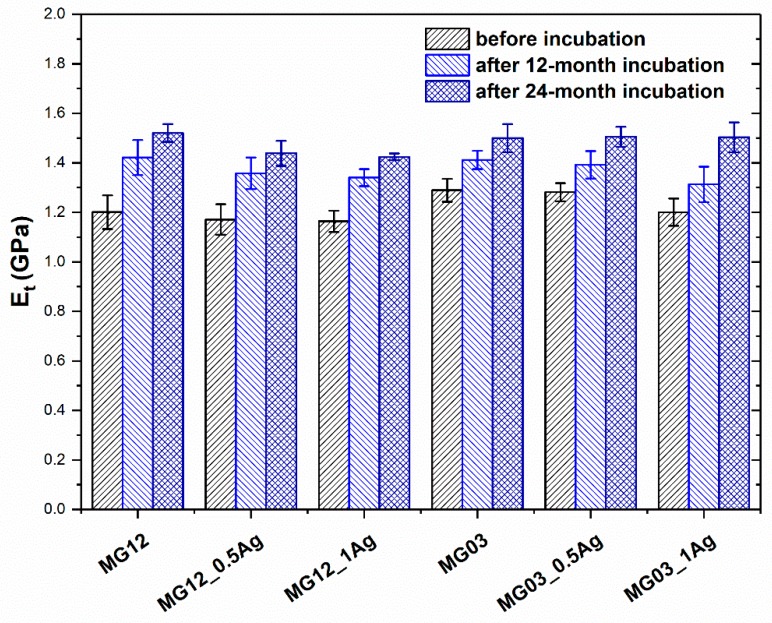
Young modulus (E) of pure polymers and polymers containing 0.5 and 1.0 wt. % of silver nanoparticles (AgNPs).

The tensile strength and Young modulus of polymer matrices and PP/AgNPs composites were determined in mechanical tests (Figure 6 and Figure 7). The 0.5 and 1 wt. % addition of silver nanoparticles did not affect the strength or the Young modulus value for either type of the polymer matrices. On the other hand, the incubation process in the aquatic environment increased both the tensile strength and Young modulus. In particular, a visible change was noted for the Young modulus, as its value increased with the increasing content of silver nanoparticles. The observed phenomenon concerned both the MG03 and MG12 polymer. The Young modulus for the MG03 matrix was 1.3 GPa, and for the MG12 matrix it was 1.2 GPa. After the 12-month incubation, the Young modulus for the MG03 and MG12 matrix and their composites was 1.4 GPa, and after 24 months of incubation it was1.5 GPa. With respect to the tensile strength, the initial value *σ*_M_ for the MG03 and MG03_0.5Ag materials was 36 MPa, for the MG03_1Ag composite it was 35 MPa, while for the MG12 matrix it was 34 MPa and for its composites it was 32 MPa. After 12 and 24 months of incubation, the strength of the composites increased to 42 MPa. However, there was no difference in this parameter relating to the increasing content of the modifying phase.

**Figure 8:**
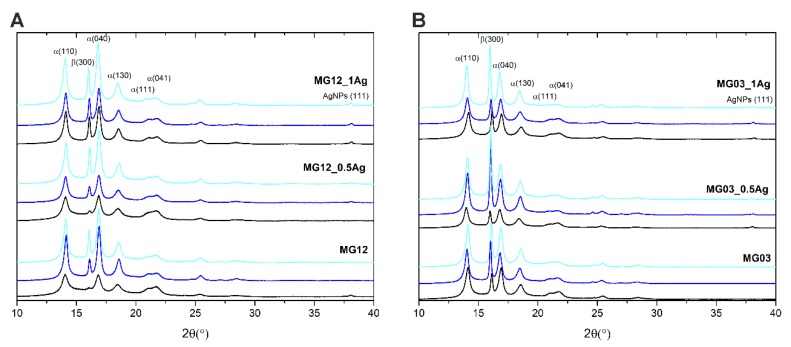
X-ray diffraction (XRD) of the pure polymers (MG12 (**A**) and MG03 (**B**)) and polymers containing 0.5 and 1.0 wt. % of silver nanoparticles (AgNPs) before incubation (black line), after 12 months (navy blue line) and after 24 months of incubation (cyan line).

The XRD tests (Figure 8) showed appearing peaks for the 2*θ* angle of 14.0°, 16.8°, 18.5°, 21.2°, and 21.6° corresponding to the crystalline α phase derived from the polymers MG12 and MG03 in the planes directions (110), (040), (130), (111) and (041), respectively. At the angle of 2*θ* = 16.0°, there was a peak corresponding to the crystalline phase β from individual polymers at the direction of planes (300). However, for the angle of 2*θ* = 38° and Miller indexes (111) there was a peak responsible for the presence of silver nanoparticles in polymer matrices. The intensity of this peak increased with the content of silver nanoparticles, still it did not change after the 12- and 24-month incubation. The XRD results also indicated an increase in the crystallinity of polymers and composites in the intensity of the peaks corresponding to the crystalline phase α and β. The peak intensity increased with the incubation time and it achieved the highest value after 24 months of incubation. The XRD studies determined the relative share of the crystalline β phase in polypropylene matrices (Table 1). The obtained results indicated that the share of the β phase increased with the content of nanoparticles in polymer matrices and with the incubation time.

**Figure 9:**
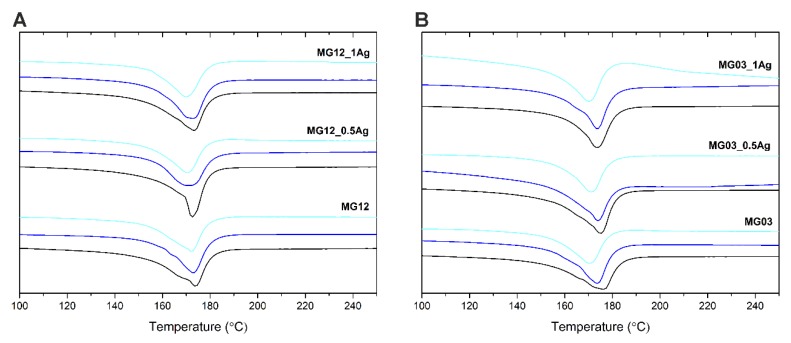
DSC of the pure polymers (MG12 (**A**) and MG03 (**B**)) and polymers containing 0.5 and 1.0 wt. % of silver nanoparticles (AgNPs) before incubation (black line), after 12 months (navy blue line) and after 24 months of incubation (cyan line).

The DSC tests (Figure 9) allowed to determine the melting temperature *T*_m_ and to calculate the degree of crystallinity *X*_C_, basing on the melting heat of the tested sample, the heat of fusion of fully crystalline polypropylene and the weight share of the modifying phase *x* (AgNPs). The results revealed (Table 1) that the melting point *T*_m_ decreased with the content of silver nanoparticles in composites. The results obtained after 12 and 24 months incubation indicated that the melting point of all the materials decreased and the fall was more significant after 24 months of incubation. The degree of crystallinity decreased with the incubation time and after 24 months the crystallinity of polymer matrices got reduced by 6.5% on average. However, for the materials containing silver nanoparticles the crystallinity was in average reduced by 12% for the 0.5 wt. % AgNPs composites and by 5% for the 1 wt. % AgNPs composites.

## 4. Discussion

Designing the polymer matrix composites modified with nanoparticles is inevitably associated with a change in the materials properties. So as to achieve the desired biological property, e.g., the bactericidal one, nanoparticles of metals, such as: gold, copper, silver, or zinc, are introduced into polymer matrices [25,26,27]. The volume introduction of nanoparticles into polymers can adversely affect the mechanical properties of composites, which is crucial in terms of implants durability and stability in the biological environment [28]. Maintaining the proper mechanical parameters at a constant level, or even strengthening the matrix, is a desirable feature. In the case of MG03_AgNPs and MG12_AgNPs composites tested after 12 and 24 months of incubation, the silver modification in the amount of either 0.5 wt. % Ag or 1.0 wt. % Ag increased the mechanical properties. The literature data revealed that low amounts of nanoadditives incorporated into a polymer matrix could improve its tensile strength and ductility [29,30,31,32,33]. For instance, 0.1–0.3 wt. % of silver nanoparticles measuring 30–110 nm increased both the elongation at break and the Izod impact strength of PP [34]. The DSC and XRD examinations also showed a change in structural properties, in particular the melting point, degree of crystallinity and proportion of the crystalline β phase. The results clearly indicated that the AgNPs addition induced the formation of the β-izotatic PP (iPP) polymorph. It was proved that the relative amount of the β form depended on the concentration of silver nanoparticles in the composites. It was proved that the β-form enhanced the impact toughness of PP at the expense of yield strength [34]. High contents of β-form of PP were obtained via the directional crystallization in a high temperature gradient field or by adding specific nucleating agents, such as calcium carbonate [2]. Silver nanoparticles also acted as effective heterogeneous nucleation agents to facilitate the PP crystallization. The heterogeneous nucleating effect of Ag nanoparticles increased the crystallization temperature of PP nanocomposites, whereas the AgNPs inclusions exhibited a nucleation effect on the crystallization of polypropylene [35]. The increasing content of nanoparticles resulted in the decrease in melting temperature and the maintained constant level or decrease in crystallinity in the composites volume. This effect was also visible after 12 and 24 months of incubation. Such a behavior pattern is consistent with the literature references. Hybiak and Garbarczyk proved that the AgNPs’ role in the iPP formation was related to the difference in the specific silver heat Ag (0.235 Jg^−1^K^−1^) and iPP (2 Jg^−1^K^−1^). The former was around 10-times lower than the latter one. During the crystallization being an exothermic process, heat from the solidifying iPP matrix was quickly absorbed by silver particles. Therefore, the temperature in the polymer around silver nanoparticles decreased [36]. They also showed that the β form occurrence might be related to the epitaxial growth of iPP on the surface of Ag or to the differences between specific heat of iPP and AgNPs. Chae and Kim indicated that silver nanoparticles in the polymer matrix could retard the heat penetration [35]. However, the SEM and surface roughness tests revealed no major differences in the surface microstructure of the materials tested after 12 and 24 months of incubation, as compared to the initial materials. The roughness tests did not show any major changes, while in the SEM observations the emerging nanosilver was noticed, which confirmed the relationship with the increasing share of the β phase determined by XRD. The incubation process in water could lead to the partial elution and exposure of silver nanoparticles located near the surface, which directly influenced the increase in the crystalline β phase. In general, the β-form crystal was less stable than the α-form crystal, which resulted in the decrease of the melting temperature [35]. It was also established that a higher content of the crystalline β phase affected the strengthening of the polymer matrix and improved elongation at break. The above phenomena were associated with the β-form occurrence in the iPP due to aggregation of AgNPs, therefore the degradation process of PP slowed down [37]. Additionally, nanoparticles in the polymer matrix retarded the heat penetration and prevented the diffusion out of the decomposed polymeric material. The changing value of surface wettability served as an indicator of polymer-based materials stability in the long-term in vitro testing. The polypropylene surface, due to the presence of CH_2_ and CH_3_ groups in its chain, had a contact angle close to 90°, which was confirmed by the results of the study. Resulting from the initial PP degradation, hydrophilic groups results were inserted into the polymer surface, thus making it more hydrophilic [38]. Furthermore, the degradation process of polyolefins could lead to the formation of chemical species containing hydrophilic hydroxyl (OH) and hydroneutral carbonyl (C=O) groups [39]. However, our results showed no significant changes in the water contact angle of the materials after the 12- and 24-month incubation in deionized water, which indicated the stability of polymer matrices. The surface wettability of biomaterials is an important surface parameter that influences the in vitro and in vivo biological response to the material. Hao et al. found that the moderate wettability (40–90°) could promote the adhesion, proliferation and osteogenic differentiation of mesenchymal stem cells [40].

## 5. Conclusions

The structural and surface properties of the PP/AgNPs composites were successfully examined. The dispersion of fillers and the composites properties depended on the filler content. The best dispersion was achieved for the composites containing 0.5 wt. % of silver nanoparticles. The incorporation of silver into polypropylene matrices improved the tensile strength and Young modulus. The values improved in accordance with the filler content increase, which was observed after the incubation. The surface properties, such as roughness and wettability, also changed accordingly to the content of the nanometric additive. The melting temperature of PP matrix in the composites containing 1.0 wt. % of silver increased by up to 1.4 °C in comparison to that of the pure PP, although the effect depended on the filler content. On the other hand, the *T*_m_ for composites with 0.5 wt. % of nanoparticles remained unaffected. The crystallinity value increased likewise, yet this effect was only observed in the surface measurements. The DSC examinations showed that the crystallinity in volume decreased with the increasing content of nanoparticles. The in vitro tests proved the mechanical and microstructural stability of the investigated samples. Both the Young modulus and tensile strength increased after the incubation. Taking into account the surface observations and XRD measurements after incubation, it can be concluded that the surface properties were altered. The conducted studies proved the suitability of all the composites for medical applications, including implants with a long-term contact in the human body.

## Figures and Tables

**Table 1:** Temperature, degree of crystallinity and relative contents of β crystals of the pure polymers and polymers containing 0.5 and 1.0 wt. % of silver nanoparticles (AgNPs).

	Before Incubation			After 12-Months Incubation			After 24-Months Incubation		
**Sample**	**DSC**		**XRD**	**DSC**		**XRD**	**DSC**		**XRD**
	*T*_m_ (°C)	*χ*_C_ (%)	*k*_β_ (%)	*T*_m_ (°C)	*χ*_C_ (%)	*k*_β_ (%)	*T*_m_ (°C)	*χ*_C_ (%)	*k*_β_ (%)
**MG12**	173.9	46.4	0.6	172.9	39.2	6.1	172.2	40.3	10.1
**MG12_0.5Ag**	172.3	47.6	1.2	171.2	38.3	7.5	170.7	36.8	11.6
**MG12_1Ag**	173.3	43.0	10.0	173.1	39.8	11.8	169.7	38.9	11.9
**MG03**	176.2	44.4	11.9	173.5	39.7	18.7	170.9	37.0	19.6
**MG03_0.5Ag**	175.2	49.3	14.7	173.9	37.8	27.5	171.2	36.6	28.6
**MG03_1Ag**	173.5	43.8	16.9	173.7	39.8	18.0	170.2	37.1	25.3

*T*_m_–melting temperature. *χ*_C_–degree of crystallinity. *k*_β_–relative contents of β crystals in the crystalline phase of PP.

## Supplementary Materials

The following are available online at https://www.mdpi.com/2073-4360/11/12/2018/s1, Figure S1: 3D confocal microscope microphotographs of pure polymers and polymers containing 0.5 and 1.0 wt. % of silver nanoparticles (AgNPs) before and 24 months of incubation.
