# Supplementary material for: Long-Lasting Examinations of Surface and Structural Properties of Medical Polypropylene Modified with Silver Nanoparticles
Source: Polymers (Basel). 2019 Dec 5;11(12):2018. doi: 10.3390/polym11122018 (PMC6960924; doi:10.3390/polym11122018)
Supplement: Supplementary file 1 [file polymers-11-02018-s001.pdf]

Supplementary materials:

Long-Lasting Examinations of Surface and Structural Properties of Medical Polypropylene Modified with Silver Nanoparticles

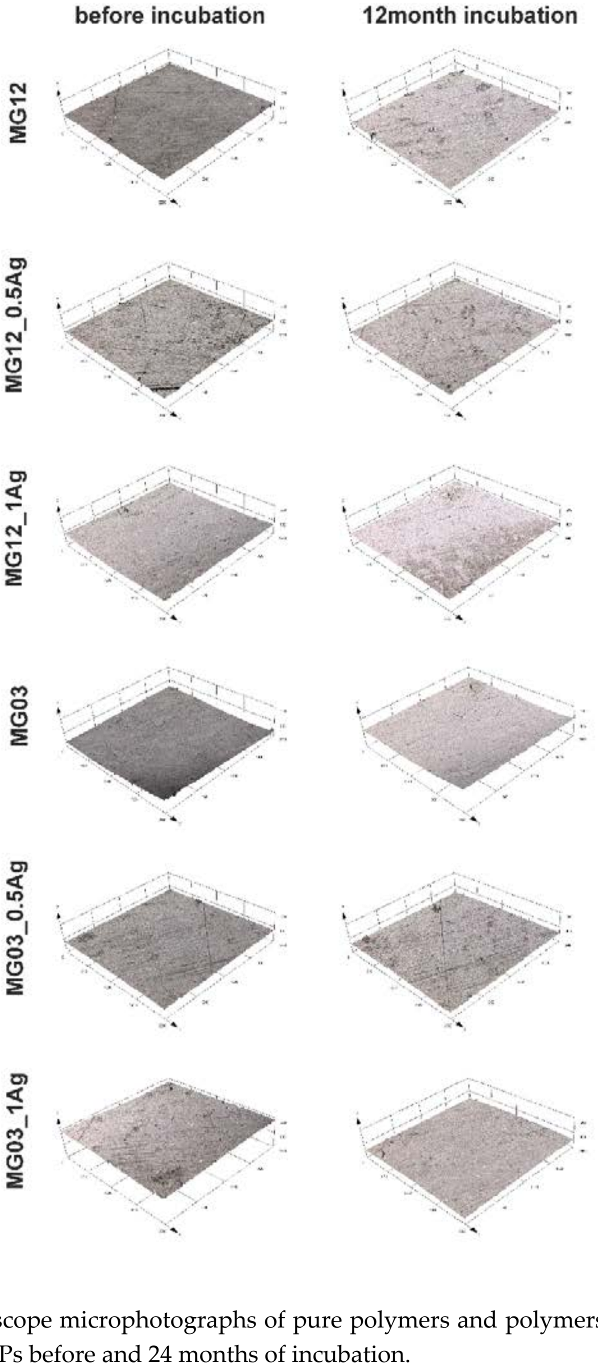

Figure S1. 3D confocal microscope microphotographs of pure polymers and polymers containing 0.5 and 1.0 wt. % of silver nanoparticles AgNPs before and 24 months of incubation.
